# Seasonal variation in food security, lifestyle, nutritional status and its associated factors of the urban poor adolescents in Kuala Lumpur, Malaysia: research protocol of a prospective cohort study

**DOI:** 10.1186/s40795-023-00680-6

**Published:** 2023-02-02

**Authors:** Janice Ee Fang Tay, Serene En Hui Tung, Satvinder Kaur, Wan Ying Gan, Nik Norasma Che’Ya, Choon Hui Tan

**Affiliations:** 1 Department of Food Science and Nutrition, Faculty of Applied Sciences, UCSI University, Cheras, 56000 Kuala Lumpur, Malaysia; 2 Division of Nutrition and Dietetics, School of Health Sciences, International Medical University, 57000 Kuala Lumpur, Malaysia; 3 Department of Nutrition, Faculty of Medicine and Health Sciences, Universiti Putra Malaysia, 43400 Serdang, Selangor Malaysia; 4 Department of Agriculture Technology, Faculty of Agriculture, Universiti Putra Malaysia, 43400 Serdang, Selangor Malaysia

**Keywords:** Food security, Urban poor adolescents, Healthy lifestyle, Climate change, Monsoon season

## Abstract

**Background:**

Climate change, obesity and undernutrition have now become a worldwide syndemic that threatens most people’s health and natural systems in the twenty-first century. Adolescent malnutrition appears to be a matter of concern in Malaysia, and this is particularly relevant among the urban poor population. Mounting evidence points to the fact that underlying factors of malnutrition are subject to climate variability and profoundly affect nutritional outcomes. Hence, it is interesting to examine seasonal variation in nutritional status and its associated factors of urban poor adolescents in Malaysia.

**Methods:**

This is a prospective cohort study following urban poor adolescents aged 10–17 years living in low-cost high-rise flats in Kuala Lumpur, Malaysia, across two monsoon seasons. The baseline assessment will be conducted during the onset of the Northeast Monsoon and followed up during Southwest Monsoon. Climate data will be collected by obtaining the climatological data (rainfall, temperature, and relative humidity) from Malaysia Meteorological Department. Geospatial data for food accessibility and availability, and also built (recreational facilities) environments, will be analyzed using the QGIS 3.4 Madeira software. Information on socio-demographic data, food security, lifestyle (diet and physical activity), and neighbourhood environment (food and built environment) will be collected using a self-administrative questionnaire. Anthropometric measurements, including weight, height, and waist circumference, will be conducted following WHO standardized protocol. WHO Anthro Plus was used to determine the height-for-age (HAZ) and BMI-for-age (BAZ). Anaemic status through biochemical analyses will be taken using HemoCue 201+® haemoglobinometer.

**Discussion:**

The study will provide insights into the seasonal effects in nutritional status and its associated factors of urban poor adolescents. These findings can be useful for relevant stakeholders, including policymakers and the government sector, in seizing context-specific strategies and policy opportunities that are seasonally sensitive, effective, and sustainable in addressing multiple challenges to combat all forms of malnutrition, especially among urban poor communities.

**Trial registration:**

The protocol for this review has not been registered.

## Background

Although many actions and efforts had been made, malnutrition in all forms, including overnutrition (overweight and obese) and undernutrition (underweight, wasting, stunting and micronutrients deficiencies), as well as diet-related non-communicable diseases (diabetes, cardiovascular diseases), continues to be alarming public health issues all over the world including both developed and developing countries [1]. According to Country Nutrition Report by Global Nutrition Report, Malaysia has been recently classified as one of the Asian countries that is currently experiencing a triple burden of malnutrition, where overweight coexists with stunting and anaemia [2]. This urges the need for urgent action to identify and address the root causes of public health issues.

As defined by World Health Organization (WHO)_,_ defines adolescents are young people aged between 10 and 19 years [3]. It is well recognized that adolescence is the vital period in the life span that experiences major anatomical, physiological, and social changes as a form of transition. During this transition from childhood to adulthood period, children begin to explore and develop their identities as a result of complex interactions with their families, communities, and culture that accompany all domains of autonomy development [4]. This dynamic period of human growth makes adolescents nutritionally vulnerable as the pubertal transition during this period is accompanied by a growth spurt where extra macronutrients and micronutrients are required [5, 6]. The burden of malnutrition can harm adolescents in multiple ways, including impaired growth, osteopenia, anaemia, retard sexual maturation, and these consequences have been shown to be associated with the early onset of chronic diseases in adulthood [7].

In this twenty-first century, climate change, obesity, and undernourishment have now become a worldwide syndemic that threatens most people’s health and natural systems globally in every part of the world [8]. While climate change requires decades or centuries to represent true shifts in the long-term means, what individuals experience in their everyday lives are simply anomalies around a stable mean, known as climatic variability [9]. Climate change is already showing significant implications for the food system and the food environment, subsequently affecting critical dimensions of food security. Thus, possess a risk to human health. People’s daily experiences with climatic variability, on the other hand, are contributing to alarming levels of malnutrition [10]. A systematic review of the existing efforts to quantify the effect of climate change on undernutrition confirmed the evidence of a significant but varied link between weather variables such as rainfall, extreme weather events, seasonality and temperature, and malnutrition at the household level [11–13]. Cumulative changes in the state of climate no matter in the short term or long term, can have profound effects on crop yields, fisheries, and livestock production. These changes in the food production system may result in economic losses and disturb the internal equilibrium food supply, resulting in food price spikes that obstruct food accessibility and affordability. Consequently, dietary patterns and food utilization have changed to adapt to these unfavourable situations. Besides, food utilization is also affected by factors such as food safety and nutritional quality aspects. These changes will hinder progress toward the eradication of malnutrition in all its forms [14, 15].

In Malaysia, the Global Food Security Index has dropped from the 28th in 2019 to 43rd in 2020, where resilience and natural resources, such as weather extreme, exposure to high-temperature rises, have been highlighted as one of the key dimensions leading to national food security deteriorates slightly [16]. Besides, urban poor communities tend to have greater exposure to natural hazards, direct dependence on climate-sensitive resources, and limited resources and capacity to adapt and cope with unexpected or immediate dangerous events [12]. Meanwhile, A study by UNICEF Malaysia [17] which focuses on urban poverty and deprivation in low-cost flats in Kuala Lumpur, reported that more than 1 in 10 children have less than three meals a day and experience undernourishment which is worse than the national prevalence [18]. The commodity inflation pressure due to reduced agricultural production might prompt a shift in their dietary patterns towards affordable yet unhealthy food supply. This raises questions about the implication of climate variability as an unprecedented confluence of pressure on food security and its associated threats to nutritional status among urban poor in Malaysia. A household study in Tanzania provides evidence that the increase in variability of temperature is associated with a decrease of about 11% in daily calorie intake [10]. A positive correlation is visible for maize and wheat prices with an increased average temperature in some countries, such as Bangladesh, Ethiopia, and Yemen [19]. Climate variability-related consequences such as a limited supply of vegetables during rainy days make vegetables more expensive and affect the overall consumption of healthful foods for this vulnerable population [12]. Hence, the occurrence of food insecurity appeared to be is one of the biggest problems among this population, and this forced the community to have a monotonous diet with poor nutrition, which over time can constrain children and adolescent’s growth and development [10, 20]. Without action, the likely increased risk of food insecurity and malnutrition brought on by climate change and variability are shouldered by most disadvantaged urban poor children and adolescents [20]. In addition, climate change, food security, and malnutrition are now commonly regarded as high-priority objectives for many countries in terms of sustainable development, as recognized by the UN Sustainable Development Goals (SDGs) [21].

Climate-related effects on malnutrition could also be reflected in food utilization and associated with the nutritional quality of food, its production and consumption, and food safety. A recent systematic review and meta-analysis by Stelmach-mardas et al. [22] that assessed the effect of season on food intake and total energy intake confirmed that season is a potential determinant of the intake of fruits, vegetables, eggs, meat, cereals, and alcoholic beverages and that an increase in energy intake was observed during winter or post-harvest season. In tropical countries such as Cambodia, the consumption of rice, starchy roots, vegetables and products, condiments, and spices tend to be higher during wet seasons, while more alcoholic and non-alcoholic beverages were consumed more during the dry seasons [23]. Another study conducted in the North of Ghana examined the effects of seasonal variation on dietary diversity was notable, especially in the consumption of vitamin A-rich fruit and vegetables where dark green leafy vegetables were consumed more during rainy seasons while deep yellow, orange, and red vegetables were consumed more during dry seasons [24]. Increased inter-seasonal climate variability thus intensifies nutrient intake fluctuations, exacerbating negative effects on nutrition. This is supported by the prevalence of seasonal variations in childhood malnutrition. A longitudinal study conducted in east rural Ethiopia found that higher prevalence of acutely malnourished children in the dry season compared to rainy seasons [25]. Contrarary On the other hand, the Ghana study reported better height-for-age and body mass index-for-age during the dry season compared to the wet season [26].

Another possible link between climate variability and malnutrition, specifically obesity, is the reduction of physical activity. Past research reported that when the weather is too cold or too hot to go outdoors, even just for a walk or a jog can simply reduce physical activity levels [27]. Previous systematic review and meta-analysis studies suggested that children’s engagement in physical activity varies by season, whereby healthier physical activity level was observed in Spring compared with other seasons [28]. A study in the US reported that adolescents were less physically active during winter and on rainy and short sunlight days, while higher physical activity during warmer and rain-free days [29]. This further confirmed that climate variability does significantly influence the level of physical activity.

To address key issues of malnutrition, environmental exposure in relation to food and physical activity should also be considered as a part of key determinants. A neighbourhood environment that promotes unhealthy dietary intake and a sedentary lifestyle can lead to an increase in malnutrition [30]. In the increasingly urbanized world, cities dwellers are facing challenges of having limited or difficult access to healthy food markets, known as ‘food deserts’ or confronted with an abundance of unhealthy food that is high in calories, low in nutrients, and processed foods, known as ‘food swamp’. These phenomena are particularly more common among urban communities that face a high degree of poverty [31]. The lack of availability and access to nutrient-dense food may have serious adverse consequences on the nutrition and well-being of children and adolescents [31, 32].

Similarly, built environments, including the combinations of coexisting stimuli such as recreational facilities, green spaces, parks, land use, and transport infrastructures, influence an individual’s ability to achieve the recommended level of physical activity. Urbanization had to cause the reduction of physical activeness due to the urban environmental quality and the setting [33], whereby high-density traffic, limited sidewalks, parks, and recreational facilities as well as overcrowding and safety in the neighbourhood such as crime and violence are commonly reported as the significant barriers that could discourage, or crease active living and causing changes in lifestyle behaviour [34, 35]. Other important environmental variables include mixed land use, transportation system, and the density of built spaces which are related to the walkability of a neighbourhood, consequently, weight status [36].

As Malaysia is located close to the equatorial line, hence no extreme weather changes can be observed and are free from inter-seasonal variability as seen in four-season countries [10]. However, the climate system of Malaysia is largely influenced by the Southeast Asia Maritime Continent monsoon, which consists of two monsoon regimes, Southwest Monsoon (summer monsoon, late May to September) and Northeast Monsoon (winter monsoon, November to March) [37, 38]. With this, it is hypothesized that seasonal variation in food security, dietary consumption, and physical activity behaviours that leads to nutritional changes, as observed in the four-season countries, may also be observed in Malaysia during the two monsoon seasons. Nevertheless, these anticipated changes and their long-term effects were rarely studied in Malaysia. Additionally, most of the research on seasonality are generally focuses on young children [24, 25, 39, 40] and adults [22, 23], and adolescents are often understudied despite their apparent vulnerability is observed. With this, It is unclear of the possible long-term impact of climate variability on the changes in nutrition-related components in Malaysia. This makes the need to study the effects of seasonal variability on malnutrition and its associated factors especially among the urban poor adolescent population in Malaysia.

## Methods

### Study aim

The main aim of the study is to examine the seasonal variation in food security, lifestyle factors, neighbourhood environmental factors, and nutritional status of the urban poor adolescents in Kuala Lumpur, Malaysia. To achieve the aim, the specific research objectives to be investigated in the study are as follows:

- To determine the food security, lifestyle factors, neighbourhood environmental factors, and nutritional status of the urban poor adolescents in Kuala Lumpur, Malaysia.
- To determine the seasonal differences in food security, lifestyle factors, and nutritional status among urban poor adolescents in Kuala Lumpur, Malaysia.
- To determine the effects of season on food security, lifestyle factors, neighbourhood environment, and its effects on the nutritional status of the urban poor adolescents in Kuala Lumpur, Malaysia.

### Study design

This is a cohort study that stemmed from two cross-sectional studies that will be carried out during the onset of the Northeast Monsoon season and will be followed up during the Southwest Monsoon season to capture a different season. Recruitment of the subjects will begin upon obtaining ethical clearance from the Institutional Ethics Committee of UCSI University [Reference code.: IEC-2021-FAS-03)] and permission to conduct the study from Kuala Lumpur City Town Hall (DBKL) [Reference code: DBKL/JPKKB/SPZ/29–3 Jld 13 (48)].

### Study setting and sampling method

**Fig. 1:**
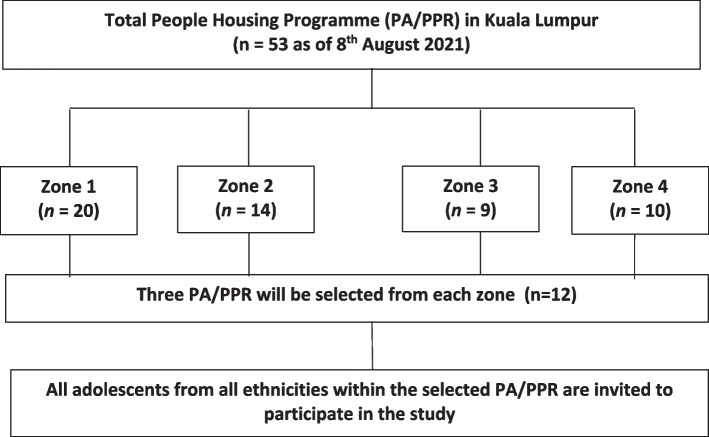
Multistage stratified random sampling method of the study

**Fig. 2:**
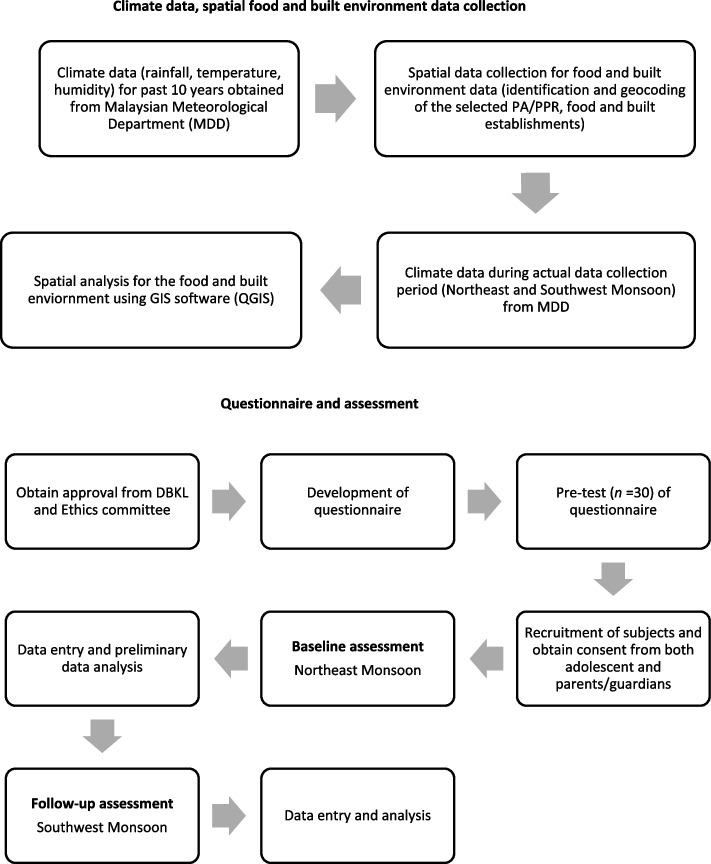
Data collection procedure

This study followed a multistage stratified random sampling design **(**Fig. 1**)**. First, a complete list of the People’s Housing Programme (*Perumahan Awam, PA or Program Perumahan Rakyat, PPR*) (*n* = 53), located in Kuala Lumpur is obtained from the Kuala Lumpur City Town Hall (*Dewan Bandaraya Kuala Lumpur, DBKL*) and further stratified into four zone areas based on the zone office management by DBKL. Subsequently, three flats from each zone area will be randomly selected (*n* = 12). A convenience sampling method will be applied for the recruitment of the subjects whereby the residential president of PA/PPR shared e-flyers through social media platforms with the residents for the recruitment of the adolescents via the parents. In addition, adolescents will be also approached by door-to-door recruitment methods at all the residential blocks. All adolescents from all ethnicities within the selected flats are invited to participate in the study. The purpose and procedures of the study will be explained to the adolescents and their parents/guardians. A detailed information sheet will be provided for a better understanding of the study. Parents/guardians will be requested to sign a written informed consent to allow their child’s participation, and assent will be obtained from adolescents to indicate their willingness to participate before the commencement of the study. Data collection will be conducted by enumerators who have been trained, monitored, and supervised throughout the study. Each adolescents will be given a unique identification number to ensure anonymity. The flow diagram of the study is depicted in Fig. 2.

### Inclusion and exclusion criteria

There are a few inclusions and exclusion criteria set for the adolescents to be recruited and qualified to participate in this study. The criteria are as below:

#### Inclusion criteria

- Malaysian adolescents aged between 10 to 17 years old.
- Adolescents currently living in low-cost high-rise flats in Kuala Lumpur at least for the past 12 months.
- Healthy adolescents with no physical and learning disabilities, as confirmed by parents.

#### Exclusion criteria

- Adolescents who are under any special diet or diet restriction.
- Adolescents who have chronic medical problems such as heart disease, diabetes, cancer, etc.
- Adolescent females who are pregnant or lactating.
- Adolescents who were infected with COVID-19 at the point of the study.

### Sample size calculation

The sample size will be calculated for the baseline cross-sectional analysis of this study. The sample size is determined using single proportion formula [41] for estimating prevalence. Based on the proportion of adolescents who were obese in Kuala Lumpur, Malaysia (15.8%) [14], with the desired level of confidence at 95% and desired level of precision of 0.05, a minimum of 204 subjects are required for the study. In consideration of the non-response rate and attrition rate among adolescents, the sample size is increased (+ 25%) to 256 adolescents.

### Measurements

Climate data will be collected from Malaysian Meteorological Department (MMD) to confirm the variation in meteorological parameters. Spatial food and built environment data will be collected using geographic information system (GIS) for urban poor adolescents residing in PA/PPR in Kuala Lumpur, Malaysia. Two self-administrated Malay language questionnaires, a parental questionnaire and a adolescent questionnaire, will be developed and pre-tested. All parents and adolescents will be given instructions and explanations of the questionnaire variables. Anthropometric, physical fitness level, and haemoglobin level assessment will be conducted after the consent form and questionnaire are filled up. To ensure the accuracy of the data collected, participants will be re-interviewed and double check of questionnaires. Table 1 shows the details of the variables assessed during baseline and follow-up in this study.

**Table 1:** Summary of data collection and timeline

Study variables	Baseline assessment (Northeast Monsoon)	Follow-up assessment (Southwest Monsoon)
**Parental Questionnaire**		
Parental/Guardian’s information		
Age	*	
Ethnicity	*	
Marital status	*	
Educational attainment	*	
Occupation	*	*
Monthly household income	*	*
Adolescents’ information		
Age	*	
Date of birth	*	
Sex	*	
Ethnicity	*	
Educational Attainment	*	
Household size	*	
Number of school-going children	*	
Duration of living in PA/PPR	*	
Medical illness or disabilities	*	
Medication or supplement use	*	
18-item USDA Household Food Security Module	*	*
Perceived Nutrition Environment Measures Survey	*	*
Physical Activity Neighbourhood Environmental Survey	*	*
**Adolescent’s Questionnaire and Assessment**		
Pubertal Development Scale	*	*
2-day diet recall	*	*
Eating Behaviours Questionnaire	*	*
Physical Activity Questionnaire for Older Children	*	*
Screen time	*	*
Step counts	*	*
Changes in lifestyle-related behaviour during COVID 19 pandemic	*	
Modified Harvard step test	*	*
Body weight	*	*
Height	*	*
Waist circumference	*	*
Body fat percentage	*	*
Haemoglobin level test	*	*

#### Climate data

Climate data will be assessed through the monthly mean data for meteorological parameters, including rainfall (mm), temperature (°C), and relative humidity (%) from the Malaysian Meteorological Department from the monitoring stations located in Petaling Jaya, FRIM Kepong, KLIA Sepang, Subang, and Hulu Langat. Climate data during the actual data collection period will be assessed for both Southwest and Northeast monsoon seasons. Besides, climate data of selected meteorological parameters for the past 10 years will also be extracted to identify the climate trend for both monsoon seasons and to confirm any climate variation over the years.

#### Spatial food and built environment

Spatial food and built environment are defined using a buffer zone area of defined geographic distance from the low-cost flats. QGIS 3.4 Madeira will be used to generate walking-distance circular buffers for a distance of 1000 m on-street network away from residential flats. These are the common buffer sizes applied in active research transport as this is the distance that can be covered by a 10–15-minute walk [42]. The exact address of each selected flats is required, which will be converted into geographic coordinates. The spatial data of all food and built establishments within the residential neighbourhood environment are geocoded to obtain latitude and longitude, where on-site data collection will be performed using a Qmini A7 High-precision (country that produce it) GIS handheld device. Types of food establishments included are supermarkets (e.g., AEON, GIANT, TESCO, large local wet markets, etc.), fast-food restaurants (e.g., KFC, Pizza Hut, McDonald’s, Texas Chicken, etc.), restaurants, convenience stores (e.g., 7-eleven, KK mart, Shell Mart, etc.), street food and specialty food stores (e.g., bakeries, fruit and vegetables, gourmet, meat and fish markets). Built establishments for physical activity included parks and public recreational facilities (e.g., soccer fields, basketball courts, community centers, pools, and playgrounds). Besides, the location of bus stops and the station will also be collected. The assumption is made that no substantial changes in the distribution and number of this food and built establishments throughout the study.

Using the geocoded data, the following spatial neighbourhood food and built environment indicators will be created: (1) density of food and built establishments within 1000 m network buffer zones; and (2) proximity to food and built establishments (closest distance, via the street network, from PA/PPR to each type of food and built establishment). The density and proximity to the food and the built establishments will be determined in QGIS software using the Buffer, Mapping, and Count Point analysis tools.

#### Adolescent’s questionnaire and assessment

##### Physical development

Pubertal Development Scale (PDS) will be used to assess the pubertal status of adolescents [43, 44]. A total of five items are assessed in PDS to rate the growth spurt, body hair growth, and skin changes using a four-point scale: “not yet started” (1), “barely started” (2), “definitely started” (3), “seems complete” (4), and “I don’t know” (0). On a similar scale, boys will be asked to rate their development of facial hair and voice change, whereas girls rated their breast development and whether they have reached menarche, present (4) and absent (1). Based on the sum of the scores, adolescents will be categorized into pre-pubertal, early pubertal, mid-pubertal, late pubertal, and post-pubertal [43, 44].

##### Physical activity and fitness level

Physical activity level will be assessed subjectively using the physical activity questionnaire for older children (PAQ-C) [45]. The Malay version of PAQ-C had been validated among adolescents aged 10–17 years old [46]. A total of 10 items are assessed in the PAQ-C to assess the general levels of physical activity of adolescents over the past seven days and will be scored based on a 5-point scale ranging from ‘no activity’ (1), and ‘7 times or more’ (5). The mean total physical activity score will be calculated and classified into low (1–2.33), moderate (2.34–3.66), and high (3.67–5) [47]. Additionally, screen time such as watching television, using a computer, and playing video games of an adolescent during weekends and schooling days will be assessed as well and further classified into screen time less than two hours and more than two hours [14].

In addition, a subsample of 50 adolescents will be randomized for objective measurement of physical activity level using a pedometer (YAMAX Digi-Walker SW-700) [48]. Adolescents are assigned a pedometer, elastic belt, and a 4-day pedometer diary. The pedometer will be distributed on the same day of data collection and adolescents are instructed to self-monitor their physical activity for four consecutive days with at least one weekend day [49] by wearing the pedometer at the waist from the time they wake up until they go to bed at night, excluding water-based activities and bathing time. Subjects are requested to record the day-end values for pedometer steps, other readings displayed, and non-ambulatory activities (e.g., swimming, cycling, etc.) and to reset to zero every day. A reminder will be sent to the subjects or their parents/guardians at least once throughout the data collection period.

The physical fitness test will be conducted using a modified Harvard Step Test [50]. This method was used in previous local studies [51, 52]. Subjects are requested to wear light clothes in order to carry out the test. Subjects are instructed to step up and down on a 30 cm high step box with both feet for a maximum of five minutes or until fatigue compelled him or her to stop and rest. Immediately, the heart rates at zero, one, and two minutes of rest will be measured using an automatic blood pressure monitor (OMRON HEM-7120 Automatic Blood Pressure Monitor, OMRON HEALTHCARE Co., Kyota, Japan) and recorded, as well as the total duration of exercise. Physical fitness scores will be calculated and classified into “unacceptable” (< 65), “marginally acceptable” (65–79), and “acceptable” (≥80) [50].

##### Dietary assessment

A two-day 24-hour dietary recall which comprised one weekday and one weekend, is used to determine the food consumption and nutrients intake of subjects. The dietary recall method is carried out through face-to-face interviews. Subjects will be asked to recall and report foods and beverages consumed. Detailed information including types, brand names of commercial products, food preparation method as well as the amount consumed with the aid of a set of standard household measurements such as spoons, teaspoons or tablespoons, cups, glasses, bowls, and plates, along with the use of the Food Atlas book [53]. Dietary intake data will be analyzed using the Nutritionist Pro™ Diet Analysis Software Version 4.0 (Axxya Systems, Stafford, TX, USA) based on the food listed in the Malaysian Composition Database Nutrient Composition of Malaysian Food. This was then further evaluated for the overall diet quality of the subjects using the Standardized Malaysian Healthy Eating Index (S-MHEI) [54].

The S-MHEI consisted of 11 components, including eight food groups (total grains, whole grains, fruits, vegetables, meat/poultry/eggs, legumes/nuts, and milk/milk products) and three nutrient groups (total fat, sodium, and sugar). The scoring of these components is calculated based on the serving size and nutrient intake recommended by the Malaysian Dietary Guidelines [55] and Malaysian Dietary Guidelines for Children and Adolescents [56]. The score of each component ranged from 0 to 10, which is calculated proportionately for the in-between whole-number responses. However, as total grains and whole grains are from the same food group, the maximum score provided to each component is five to avoid scoring overlap. The total S-MHEI score is obtained by summing up the score of each component with a score range of 0 to 100. A higher S-MHEI score indicates better diet quality. Based on the maximal dietary intake score of 100, the subjects will be categorized as ‘good diet quality’ (> 80%), ‘diet quality needs an improvement’ (51–80%) and ‘poor diet quality’ (< 51%) [54].

The frequency of meal consumption will be assessed using six items from the Eating Behaviours Questionnaire (EBQ) [57]. Adolescent is required to indicate the frequency of each meal consumption and snacking behaviour, including breakfast, morning snack, lunch, evening snack, dinner, and supper, based on an 8-point scale ranging from never (zero) to every day (7 times) in a week.

##### Changes in lifestyle-related behaviour during the COVID-19 pandemic

The 20 items short questionnaire developed by Kumari et al. [58] will be used to assess the changes in lifestyle-related behaviour of adolescents during the COVID-19 pandemic. Subjects will be asked in assessing the changes in dietary habits (intake, meal pattern, and snack consumption), physical activity (duration and type), and sleep pattern (duration and quality), using a 5-point Likert scale ranging from “significantly decreased” (+ 2) and “significantly increased” (− 2). It is noted that some of the items (Item 4–5, 11–16, 19) are reverse scored. Besides, items 3 and 18 are scored as “grossly similar” (0), “slightly increased/decreased” (− 1), and “significantly increased/decreased” (− 2). The total score will be calculated and a higher score indicating shifting toward a healthy lifestyle during the COVID-19 pandemic.

##### Anthropometric measurements

All equipments will be calibrated before the measurements are performed. The body height of subjects will be measured using a calibrated vertical stadiometer (Seca 213 portable stadiometer, SECA GmbH & Co., KG. Hamburg, Germany), whereas weight and body fat percentage will be measured using the Bioelectrical Impedance Analyzer (OMRON HBF-375 Karada Scan Body Composition Monitor, OMRON HEALTHCARE Co., Kyota, Japan). Both height and weight measurements are recorded to the nearest 0.1 cm and 0.1 kg, respectively. The nutritional status of adolescents will be determined based on Z-scores for height-for-age (HAZ) and BMI-for-age (BAZ) of WHO growth reference 2007 for adolescents between 5 and 19 years old using WHO AnthroPlus version 1.0.3 software (WHO, Geneva, Switzerland). The classification as followed, stunting (HAZ < -2SD), normal height (HAZ > -2SD), thinness (BAZ < -2SD), normal weight (−2SD ≤ BAZ ≤ +1SD), overweight (+1SD < BAZ ≤ +2SD), and obesity (BAZ > +2SD) [59]. The body fat percentage will be classified using percentile scores for sex and age based on the findings by McCarthy et al. [60] into underfat (<2nd percentile), normal (2nd – 85th percentile), overfat (>85th – 95th percentile), and obese (>95th percentile).

Waist circumference will be measured at the midway between the lowest rib and the superior border of the iliac crest as recommended by WHO [61] using a non-elastic SECA 201 Ergonomic circumference measuring tape (DECA GmbH & Co., Hamburg, Germany) to the nearest 0.1 cm. A cut-off point of the 90th percentile of waist circumference percentile classification for Malaysian children and adolescents [62] will be used to identify abdominal obesity. For adolescents aged 17 years and above, WHO/IASO/IOTF (2000) for Asian cut-off [63] will be used instead. Abdominal obesity was defined as ≥90 cm for men and ≥ 80 cm for women. Mean average values will be used for data analysis.

##### Anaemia status assessment

A haemoglobin level test will be performed to identify the anaemia status of each adolescent by using the HemoCue haemoglobinometer (HemoCue® Hb 201+ System, Angelholm, Sweden), point-of-care testing on capillary blood samples. Anaemia status and level of severity are determined according to the age and gender-specific haemoglobin cut-off point as defined by WHO [64]: (i) < 115 g/L for 5 to 11 years of age; (ii) < 120 g/L for 12 to 14 years of age and non-pregnant women (15 years of age and above); (iii) < 130 g/L for men (15 years of age and above).

#### Parental questionnaires

##### Sociodemographic background

Sociodemographic data will be collected for both adolescents and parents using a self-administrated questionnaire. Adolescent information includes age, sex, date of birth, ethnicity, household size, number of school-going children, duration of living in PA/PPR, medical illness or disabilities, medication, and supplement use, whereas parental/guardian information includes age, educational attainment, marital status, occupation, and monthly household income.

##### Household food security status

Food security status is measured using the United States Department of Agriculture (USDA) 18-item Household Food Security Survey Module (HFSS) which captures all severity levels of household food security and children’s condition in the household [65]. This section of the items will be filled by the parents or the guardians as parents or guardians will be responsible for purchasing or preparing the food for the household and the adolescent. A total of 18 items will be asked and answers of “yes”, “often true”, “sometimes true,” “almost every month,” and “some months but not every month” are coded as affirmative (1 point). The sum of affirmative responses to the 18 questions is referred to as the household’s raw score. With a maximum score of 18, subjects will be categorized into high food security (0), marginal food security (1–2), low food security (3–7), and very low food security (8–18). Subjects will be further dichotomized into two groups, the food secure group (high or marginal food security) and the food insecure group (low or very low food security) [65].

##### Perceived food environment

Perceived Nutrition Environment Measures Survey (NEMS-P) by Green and Glanz [66] will be used to assess the parents’ perceived food environment. The NEMS-P core components that measured community (seven items), consumer nutrition environment (24 items), and home food environment (seven items) will be examined. Composite scores will be calculated for each domain.

Community nutrition environments assess the store and restaurant accessibility, including mode of travel to the store, distance travelled to store/restaurant, as well as store motivation (importance of store proximity to home and other places where time is spent) and restaurant motivation (importance of convenience).

Consumer nutrition environments can be further categorized into store consumer and restaurant consumer nutrition environment. Store consumer nutrition environments include 17 items on store availability of healthy food choices, store motivation, price of fruits and vegetables, placement or promotion of unhealthy/healthy items, and nutrition information. Subjects will be asked to indicate their level of agreement on the statements using a 5-point scale ranging from “strongly disagree” (1) through “strongly agree” (5). Store motivation such as the importance of selection, quality, and price of food is assessed using a 4-point scale ranging from “not at all important” (1) through “very important” (4). Pricing for fruits and vegetables is assessed using response choices of very expensive (1) to very inexpensive (4). For the restaurant consumer nutrition environment, a total of seven items will be assessed on the availability of healthy options, promotions, and the cost of healthy options at restaurants using a 5-point scale ranging from “strongly disagree” (1) through “strongly agree” (5).

The home food environment includes items on the availability and accessibility of healthy and unhealthy food in the home. Subjects will be asked to indicate the availability of fruits/vegetables, healthy food, and unhealthy food based on the given list. Besides, the accessibility of healthy and unhealthy food will be assessed using a 4-point scale of “never or rarely” (1) to “almost always” (4).

##### Perceived built environment

Physical Activity Neighbourhood Environmental Survey (PANES) by Sallis et al. [67] will be used to assess the parents’ perceived environmental support for physical activity. A total of 17 questions will be asked in measuring the attributes of neighbourhood built and social environments hypothesized or known to be related to physical activity using a 4-point Likert scale ranging from “strongly disagree” (1) through “strongly agree” (4). The items can be categorized as residential density (item 1), land use mix (items 2 and 17), transit access (item 3), pedestrian infrastructure (items 4 and 13), bicycling infrastructure (items 5 and 14), recreational facilities (item 6), street connectivity (item 12), crime safety (items 7 and item 16), traffic safety (items 8 and 15), pedestrian safety (item 9), aesthetic (item 10). The mean total score will be calculated and the higher the score, the higher the perceived neighbourhood environment support for physical activity.

### Data management and analysis

Data analysis will be performed using IBM SPSS 25.0 (SPSS Inc., Chicago, IL, USA). A test of normality will be performed using a skewness value (between − 2 and + 2) for the continuous variables. The univariate analysis will be used to report descriptive data including mean score, median, and standard deviation for continuous variables and percentage and frequencies for categorical variables. Independent-samples *t*-test and One-way ANOVA will be used to compare differences between groups for continuous variables and chi-square analysis will be conducted to examine associations for categorical variables. Correlation test will be used to see if correlation exist between variables. A paired-samples *t*-test will be conducted to determine the statistical differences between the two monsoon seasons. Logistic regression will be performed separately for seasons to determine the relationship between nutritional status and selected variables (Food security, lifestyle factors and neighbourhood environment factors). The Generalized Linear Mixed Model will be used to determine the seasonal effects on food security, lifestyle factors, neighbourhood environmental factors, and its effects on nutritional status. The significance level for all analyses is set at *p* < 0.05.

### The status and timeline of the study

The first adolescent was enrolled on the 1st of November 2021. The recruitment process and baseline data collection were completed on the 30th of April 2022. At the time of submission, all de-identified subjects data are kept and manage but have not yet been processed for analysis.

## Discussion

This study is intended to provide a detailed seasonal analysis of changes in nutritional status and its associated factors, including food security, lifestyle-related and neighbourhood environmental factors of urban poor adolescents in Kuala Lumpur, Malaysia. The valuable findings of this study hoped to enlighten and enable relevant stakeholders, including policymakers and the government sector to better understand the seasonal nature of malnutrition in Malaysia and to seize appropriate programme and policy opportunities that are seasonally sensitive, effective, and sustainable in addressing multiple challenges to combat all forms of malnutrition, also known as double-duty actions for nutrition. Information gathered in this study may be also useful in the future prediction of possible outcomes in relation to the seasonal variation and climate changes in the region. On the other hand, this study will also provide insights into household food security, diet quality, physical activity, and physical fitness levels of urban poor adolescents, which can be used as references in the formulation of future nutrition initiatives to improve nutrition and health status of urban poor communities in Malaysia. Evidence-based nutrition recommendations could aid in strengthening existing policies and measures that are relevant to adolescent nutrition by promoting healthy dietary habits and physical activity while also increasing resilience to food insecurity.

This present study has several strengths as this is the first study that examines the underlying factors of malnutrition that are subjected to seasonality and affecting the nutritional outcomes of urban poor adolescents. Assessments at both time points across the monsoon allow the assessment of the change in nutritional status and associated factors. Multiple assessments of nutritional status, including weight status, body composition, and haemoglobin status, could provide a more comprehensive understanding of the nutritional status of urban poor adolescents, which these data are scarce among the urban poor population in Malaysia. Besides, using both objective (spatial) and subjective (perceived) assessments to assess the food and built environment provides a complete picture of the residential neighbourhood environment of the urban poor. In addition, a better understanding of both physical and perceived food and the built environment surrounding urban poor communities could help to develop an effective system approach that mitigates neighbourhood environmental consequences of malnutrition by ensuring universal access to healthy food sources and supporting infrastructure in the urban poor areas.

In terms of limitations, the study results may not be representative of the general population of adolescents in Malaysia as this study is designed to focus only on the urban poor adolescents who are residing in low-cost high-rise flats in Kuala Lumpur, Malaysia. A nationwide study could be implemented in a different residential setting to ensure the generalizability of the study outcome. Besides, since the study is being conducted during the COVID-19 pandemic period, which could increase drop-out rates. To address these issues, researchers will ensure that the data collecting process complies with the standard operating procedure (SOP) that has been established by relevant authorities while also thoroughly explaining the data gathering process to parents and adolescents. Moreover, additional effects of the COVID-19 pandemic on the study variables, such as food security status, financial status, food environment, and diet may have been overlooked in this study, requiring further investigation.

## Data Availability

Data analyzed or generated in the study will be available upon reasonable request from the corresponding author.

## Abbreviations

ANOVA: Analysis of Variance
BMI: Body Mass Index
DBKL: Kuala Lumpur City Hall (*Dewan Bandaraya Kuala Lumpur*)
EBQ: Eating Behaviours Questionnaire
GIS: Geographic Information System
HLPE: High-Level Panel of Experts
MOH: Ministry of Health Malaysia
NEMS-P: Perceived Nutrition Environment Measures Survey
NPANM: National Plan of Action for Nutrition of Malaysia
PA/PPR: People Housing Programme (*Perumahan Awam/ Program Perumahan* Rakyat)
PAQ-C: Physical Activity Questionnaire for Older Children
PANES: Physical Activity Neighbourhood Environmental Survey
PDS: Pubertal Development Scale
SDG: Sustainable Development Goals
S-MHEI: Standardized Malaysian Healthy Eating Index
UN: United Nations
USDA: United States Department of Agriculture
WHO: World Health Organization
